# Composting for a More Sustainable Palm Oil Waste Management: A Systematic Literature Review

**DOI:** 10.1155/2022/5073059

**Published:** 2022-11-10

**Authors:** Jajang Supriatna, Mieke Rochimi Setiawati, Rija Sudirja, Cucu Suherman, Xavier Bonneau

**Affiliations:** ^1^Agricultural Science, Faculty of Agriculture, Padjadjaran University, Bandung 45363, Indonesia; ^2^ANJ Research Center, PT. Austindo Nusantara Jaya Tbk., Belitung 33561, Indonesia; ^3^Soil Science, Faculty of Agriculture, Padjadjaran University, Bandung 45363, Indonesia; ^4^Agronomy Department, Faculty of Agriculture, Padjadjaran University, Bandung 45363, Indonesia; ^5^Perennial Crops Department, AbSys Research Unit, Centre de Coopération Internationale en Recherche Agronomique pour le Développement, Montpellier 34398, France

## Abstract

Palm oil production has increased significantly, specifically in Indonesia and Malaysia. However, this growth has raised environmental concerns due to the high discharge of empty fruit bunches, palm oil mill effluents, and other solid wastes. Therefore, this study aims to examine the treatment of palm oil waste by composting and systematically review insights into its application through a systematic literature review approach. Among the 1155 articles, a total of 135 were selected for a systematic review of palm oil waste management developments and their applications, while 14 were used for determining compost quality according to the criteria and requirements established in the systematic literature review. Moreover, using Egger's test, JAMOVI 1.6.23 software was used to analyze random effects models with 95% confidence intervals and publication bias. The results showed that palm oil waste was optimally treated by composting, which is considered as a sustainable technology for protecting the environment, human safety, and economic value. The in-vessel method with a controlled composting chamber is the best system with a minimum time of 14 days. However, it requires tight control and provides a final product with a high microbial colony form outdoors and indoors compared to the windrow system. This study is useful to see the bias of research results and helps to find new studies that need to be developed, especially in this case related to the management of palm oil waste into organic compost fertilizer and its application methods in the field. It is suggested that applying palm oil waste or compost is mainly performed by mulching. In contrast, new challenges for better processing to produce organic fertilizers and applicable technologies for sustainable waste management are recommended. The method must be affordable, efficient, and practical, combining compost quality with maximum nutrient recovery.

## 1. Introduction

Indonesia and Malaysia represent over 80% of the world's palm oil production [1]. Meanwhile, processing one ton of fresh fruit bunches (FFBs) will produce waste in the form of empty fruit bunches (EFBs) up to 0.20–0.23 ton, mesocarp fiber 0.12–0.13 ton, palm kernel shell 0.05–0.06 ton, boiler ash 0.005–0.006 ton, and palm oil mill effluent (POME) 0.77–0.84 m^3^ [2]. These amounts of untreated solid wastes potentially cause environmental problems and might reduce the competitiveness and productivity of the palm oil industry in Indonesia [3]. When the waste is not sustainably managed, it leads to land, air, and water pollution [4, 5]. For example, EFB takes a long time to degrade, and POME harms the environment by polluting groundwater and reducing soil fertility. However, if treated and managed properly, they could be a valuable resource and value-added product [6].

EFB contains several nutrients and organic matter that provide fertility to the soil and can help meet nutritional needs. A research study shows that [7] the nutritional content of N, P, K, and Mg in EFB with shredded pretreatment were 0.90%, 0.60%, 2.40%, and 0.60%, respectively. Meanwhile, when press-shredded pretreatment was carried out [7], the nutritional content was 0.80%, 0.08%, 2.01%, and 0.12%, which were generally lower for the potential contents of N, P, K, and Mg. Recycling organic materials such as EFB and factory and mill waste in solid form can reduce the use of chemical fertilizers. Consequently, the application of 40–60 tons of EFB ha^−1^year^−1^ is highly recommended to increase organic matter and fertility in less fertile soils [8].

As for POME, it also contains relatively high nutrients, especially for K of 4173 ppm and 3193 ppm, which come from POME leachate and post biogas, respectively [9], with POME pH values of 8 and 7.5, respectively, in contrast to fresh POME with a relatively acidic pH (4.33), with a relatively low K nutrient content of 446 ppm [10]. Composting is a waste management method that can treat solid (EFB) and liquid (POME) waste. For example, composting can convert EFB into valuable products for plant growth [11]. Composting is a complex biological transformation of organic matter carried out under controlled environmental conditions by a succession of microbial communities [12, 13]. There are two processes in composting, namely, aerobic and anaerobic. Composting with an aerobic system is easier to implement, and when applied correctly, it potentially reduces the volume of solid waste. This method requires sufficient oxygen supply into the solid waste pile. In contrast, the anaerobic system method tends to be more challenging to implement, but methane production is easier to control and utilize [14].

Furthermore, composting is divided into three main stages based on temperature parameters, namely, mesophilic, thermophilic, and ripening [15, 16]. During the mesophilic phase, temperature and water content increase as a signal of a rise in psychrophilic and mesophilic microorganisms that improve the biodegradation of organic compounds and takes 20 to 30 days with temperatures above 45°C to reach the maximum peak [15, 16]. Meanwhile, the thermophilic phase occurs between 30 and 100 days [15, 16] with an increase in microorganisms and as a sanitation phase in the composting process with a temperature that must be maintained from 55 to 65°C. The final phase, namely, ripening/stabilization, is the process of decreasing temperature to 30°C, which occurs after 12 weeks. This decrease is caused by the depletion of organic compounds in the compost, thereby making the C/N ratio potentially stable. The final product or compost can be used for agricultural activities.

Data of previous studies assist in determining the best steps in palm oil waste management based on the development of methods, systems, and their application. This study aims to analyze developments in the process of palm oil waste management and its application technology in the field.

## 2. Materials and Methods

Data were initially obtained from the journal published globally from 2000 to 2021 and analyzed according to predetermined topics and from collections using integrated sources such as Scopus, Google Scholar, and PubMed. The keywords used were focused on composting palm oil waste with the queries of composting palm oil, waste treatment, compost empty fruit bunch, waste management, composting process, organic fertilizer, biofertilizer, palm oil mill effluent (POME), solid waste, anaerobic digestion, biodegradation, co-composting, fertilizer application, effluent treatment, and empty fruit bunches (EFB).

**Figure 1:**
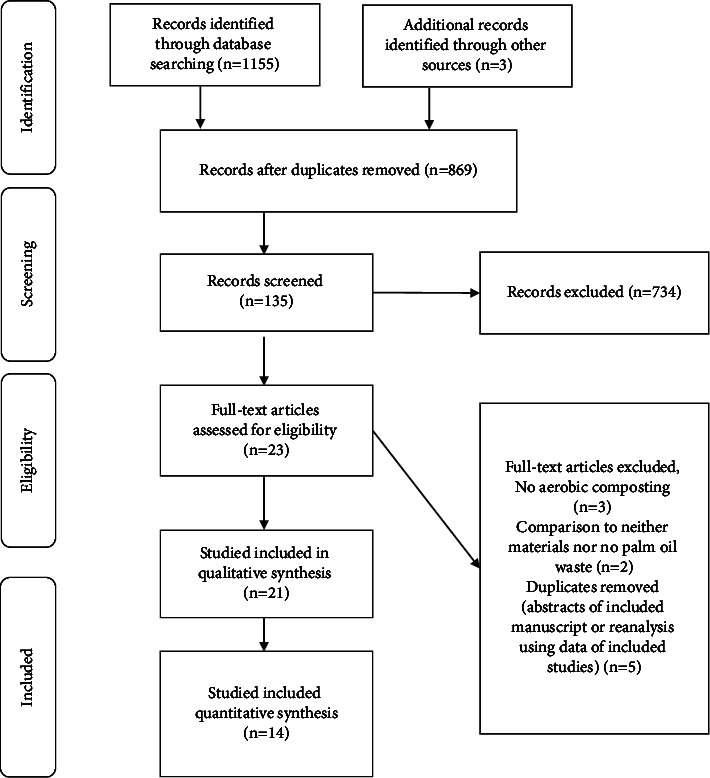
Procedure, criteria, inclusion, and exclusion for systematic review studies.

We used the PRISMA (Preferred Reporting Items for Systematic Reviews and Meta-Analyses) method to study with a systematic review [17], which is described in Figure 1. Subsequently, relevant abstracts were selected according to the predetermined requirements. We collected 869 articles, with an extensive selection of 135 articles and an intensive selection of 21 articles.

Articles were selected intensively with the following criteria: (i) the article must focus on the topic of compost from waste produced by palm oil, including EFB, POME, and other solid wastes; (ii) the article must contain experiments with a good statistical design and analysis; (iii) the results must contain variables such as composting time, humidity, temperature, pH, C-organic, N-total, C/N ratio, and metal components. The materials and methods section should be detailed enough that all procedures can be repeated. If several methods are described, it may be divided into heading subsections.

The article database was analyzed using the statistical software, namely, JAMOVI 1.6.23 [18] because it can use one of the raw data tabulated or the effect-sized value to obtain the standardised mean difference (SMD), heterogeneity, and publication bias information using the Egger test [19].

## 3. Results and Discussion

### 3.1. Definition of Compost

Compost is produced from the biological decomposition of fresh organic matter with the assistance of decomposing organisms. Plant or animal waste and microorganism residues in the form of bacteria, fungi, and algae are potential sources of organic matter for the soil because of their essential role in improving the physical, chemical, and biological properties [20].  Table 1 presents the various definitions of composting based on several opinions from books and articles.

### 3.2. Development of Palm Oil Waste Management

The traditional method of processing EFB is burning and utilizing the ashes by spreading them in the field as organic fertilizer. However, due to air pollution caused by the emission of CO_2,_ open burning is discouraged and prohibited in countries such as Malaysia and Indonesia. EFB is a lousy fuel because it contains a high moisture content of approximately 60%. Hence, it is usually air-dried to reduce 40% of the content for more efficient combustion. Moreover, using EFB for power generation requires costly preprocessing [30].

A more practical approach is to use EFB for organic mulching in palm oil plantations because of the large quantity produced. Processing one ton of FFB can produce 230–250 kg of EFB, 130–150 kg of fiber, and 60–65 kg of shell waste [2].

EFB can be composted to reduce volume and facilitate the application in plantations, thereby reducing costs. The compost can reduce volume in high amounts by up to 85% of the initial value [31]. In addition, composted EFB can increase soil C: N ratio and release beneficial micronutrients when applied to plantations as fertilizer [32]. Composting can be improved with a mixture of liquid waste of palm oil [33], decanter cake [34], and animal manure, including chicken, cow, and goat dung [35]. In general, it is a biological process that takes 2–3 months to complete, and a proper control of microbial activity is necessary to produce high-quality compost. Composting is also considered as a sustainable technology because it aims to preserve the environment and provide economic value by reducing chemical fertilizers that can cause land degradation [36].

Solid biomass waste generated by the factory contains lignocellulosic components, including large amounts of cellulose, hemicellulose, and lignin [37]. Various technologies have converted solid waste biomass in palm oil mills into value-added products. This waste can be converted into bio-oil and biochar through pyrolysis, which is the thermal decomposition of lignocellulosic biomass, such as palm oil, into gaseous and liquid fuels in the absence of oxygen [38]. This process usually occurs at a temperature of 400–600°C, and palm oil biomass pyrolysis products can be condensed into organic liquids such as bio-oil, noncondensable gas including CO, CO_2_, and hydrogen, and CH_4,_ as well as biochar. These products highly depend on the process conditions and the biomass content of cellulose, hemicellulose, and lignin. Generally, there are two types of pyrolysis: fast and slow. According to Kong et al., fast pyrolysis produces bio-oil (70%), biochar (15%), and noncondensable gas (13%). Palm oil mill produces the highest amount of bio-oil usage among solid biomass waste compared to EFB and PPF (palm pressed fiber) due to the significantly higher amount of lignin [36, 38]. Meanwhile, the highest amount of biochar can be produced when the biomass is decomposed by slow pyrolysis.

The palm oil mill waste emits a pungent odor due to the decay of the organic matter, and one of the alternative physical treatments is flotation. It aims to remove/reduce the particles in the waste water by floating the oil or fat, thereby reducing the content in the palm oil mill waste. According to Ref. [39], the flotation process of POME is very effective as a physical treatment to reduce fat or oil content, COD (chemical oxygen demand), MLSS (mixed liquor suspended solid), and MLVSS (mixed liquor volatile suspended solid) contained in palm oil mill effluent. It implies that the longer the time of the liquid in the flotation equipment, the higher the reduction [39].

Several examples of solid waste biomass, such as EFB, are processed into fibers for making paper. The paper quality obtained from EFB pulp is comparable to that of kraft hardwood [40], while the bubble slurry produced is suitable for making corrugated paper and cardboard [41]. Furthermore, EFB and palm fiber are used as fillers to reinforce polymer composites using melt blends and hot-press molding techniques. EFB can also be processed into fiber materials and used in the manufacture of fiberboard as well as medium-density fiber (MDF). Other products are coir and board fiber, cement, board, roof tile, and wood paper [42]. Recently, the isolation of cellulose nanofibers, as well as microcrystalline and nanocrystalline cellulose from EFB by acid hydrolysis, has also been demonstrated [43]. Cellulose has various applications in different industries, such as food stabilizers, pharmaceuticals, compounds, cosmetics, and as a bio-filler in composites [44]. Palm fiber contains 5% to 6% dry residual oil after CPO extraction, while the remaining oil comprises natural carotenes ranging from 4000–6000 ppm, vitamin E 2400–3500 ppm, sterols 4500–8500 ppm, and coenzyme Q10 1000–1500 ppm [45]. Most of these bioactive compounds reportedly have superior antioxidant and anticancer properties. Hence, they can reduce the risk of heart-related diseases in humans. Besides, efforts have been made to recover residual oil from palm fiber using solvent and supercritical fluid extraction [46]. In Table 2, we can see the development of palm oil waste management summarised by some references.

### 3.3. Composting Process of Palm Oil Waste

The composting process can be divided into two stages, namely, active and ripening. During the early stages, mesophilic microbes will immediately utilize oxygen and easily degraded compounds, which are then replaced by the thermophilic. Subsequently, the temperature of the compost heap increases rapidly, followed by a rise in pH until it reaches 60°C. The temperature will remain high at 40°C during the ripening phase [56], while the mesophilic microbes are then replaced by the thermophilic ones active at high temperatures. During the active decomposition of organic matter, the microbes in the compost will decompose the substrate into NH_4_^+^, CO, steam, and heat through the metabolic system with the assistance of oxygen, and the temperature reduces to the initial value. The next phase is the compost's ripening, leading to further humic complexes' formation. During the composting process, a decrease in the volume and biomass of the material occurs. This reduction can reach 30% to 50% of the initial weight, depending on the moisture content [56].

Microorganisms play an essential role in the composting process because the stability and maturity of compost depend on the microbial activity [57]. They can degrade and change constituents in the substrate, especially local types such as fungi that are useful in converting organic waste into compost [58]. Meanwhile, fungi degrade substrates containing lignin into simpler components because they have an efficient extracellular enzyme performance system, including hydrolytic and ligninolytic. Both systems produce cellulase, hemicellulase, and ligninase to degrade polysaccharides, lignin, and an open phenyl ring [59]. Several studies also stated that using rich N substances, such as palm oil mill effluent, cow or poultry dung, and rice husks, is one solution to increase the effectiveness of composting with EFB materials [35]. Other factors such as pH, humidity, and residual oil content can affect the natural composting process because they potentially interfere with the growth of fungi that act as composters.

In principle, composting is a method used to reduce the C/N ratio of the organic matter until it is equal to the level of the soil, namely, <20 or *a* = 10. It is because organic matter has a relatively high C/N ratio ranging from 58 to 60 [60]. It indicates that the higher the ratio, the longer the composting process or organic matter overhaul. The decomposition process in composting occurs under both aerobic and anaerobic conditions, as demonstrated in equation (1) in the following reaction [61]:

(1) $$\begin{array}{c} Organic\;compound+O_{2}\frac{aerobic\;microbes\;}{N,P,K}\gg H_{2}O+CO_{2}+nutrient+humus, \end{array}$$

### 3.4. Composting Method Development

The appropriate method for a given composting application is determined by a variety of factors, including location, scale of operation, the consequences of occasional odors, access to capital, the nature of the feedstocks, environmental regulations, labor availability, and business objectives.

Many composting facilities use simple methods such as passively aerated static piles and turned windrows. These methods are popular because they require minimal site modification, engineering, and capital investment. Another simple, widely used method with numerous applications is aerated static pile composting (ASP). Some composting operations have implemented advanced technology and automation methods to produce compost faster, save space, provide shelter from the elements, or reduce odors where sensitive neighbors are nearby. Several criteria can be used to label and categorize composting methods, including the mode of aeration (passive vs forced), the level of containment (open, covered, contained, or in-vessel), the degree of agitation (static vs turned), and material movement (batch vs continuous).

Table 3 summarises the comparison between the windrow-based and CECC (control environment composting chamber) systems on composting. In in-vessel anaerobic composting with CECC, EFB waste from palm oil mills will be chopped to the specified size in precomposting and mixed with liquid waste and ash from combustion. The resulting biomass mixture is then transferred by the front-end loader to the CECC and stacked into customized piles 2 meters high, 6 meters wide, and 25 meters long along the tunnel. Once filled, the tunnel will be closed and blown through a computer system to ensure the optimal conditions for the composting process according to the specified batch processing temperature. The composting process is activated using a specially formulated mixture of microorganisms mixed with a liquid waste of palm oil and sprayed onto the compost pile. The computer control system will allow the composting to be completed within two weeks with reasonable control of the oxygen level in the biomass as well as the temperature and humidity levels. At high temperatures in the composting process, a certain amount of liquid waste of oil palm is sprayed onto the compost and evaporated to maintain the optimum temperature of a humid level. After two weeks, the compost will be removed from the front-end loader from the vessel and ripened in a pile for further 14 days for preservation and cooling down.

The effectiveness of the compost in the presence of a high consistency of microbial CFU (colony forming unit) is very beneficial because it allows us to see performance signs on soil conditions, plant health, and increase in yield compared to unfortified compost produced from windrow composting mechanisms. In an environment that is not controlled by the typical windrow composting process, the composting period varies from 45 to 60 days.

### 3.5. Compost Quality Standard

The success of essential compost can be seen in the temperature parameters indicated by the mesophilic (20–40°C), thermophilic (40–60°C), and maturity (30–35°C) phases of compost with an aerobic system [16]. The results of the review showed a change in temperature from the initial phase of about 28°C to the peak phase of about 60°C and the maturity phase to 30°C of soil temperature. The thermophilic phase will be active at high temperatures to degrade hemicellulose, cellulose, lignocellulose, lignin, and lipids [63]. EFB is a high-fiber material consisting of 44.2% cellulose, 33.5% hemicellulose, and 20.4% lignin [64]. Composting is a process of decreasing organic C/N on the substrate. The high C/N ratio in compost can be caused by high holocellulose, alpha-cellulose, and lignin in organic materials used as raw materials for compost [65]. The C/N ratio is an important factor that can affect the process conditions regarding nutrients in microbes during composting and compost as a final product. The EFB composted with several other waste materials contains relatively high nitrogen and carbon, such as poultry manure, goat manure, cow manure, and palm oil mill effluent [66].

The data from the review showed that the temperature changes during the composting process are divided into the mesophilic, peak/thermophilic, and cooling/maturation phases. The mesophilic is the initial phase of composting, and it involves a temperature range of 28°C. In comparison, the activity of local microorganisms increases in the thermophilic phase at 60°C, which is the peak phase in the degradation of organic matter. Furthermore, the last phase is the compost maturity of the final product, which occurs at the range of 25°C soil temperature. Another critical factor in determining compost quality is the degree of acidity. Bacteria and fungi in the compost which are beneficial to soil and plants will breed at pH > 5.5. If the soil pH is very low, growth and development will be hampered [67]. The pH in the composting process will increase from the initial acidic state to alkaline state, but Table 4 showed that the overall pH is in the range of 7.41. This value is classified as neutral pH and complies with the requirements of SNI 19-7030-2004 and Minister of Agriculture No. 261/KPTS/SR.310/M/4/2019 (Indonesian standard). The C/N ratio obtained follows the requirements, namely, 23.42. Overall, based on the average from a total of 21 articles, the compost produced from various organic materials as well as the techniques carried out in each study is still under the requirements determined by both SNI and the Regulation of the Minister of Agriculture regarding compost, as presented in Table 4.

A total of 135 articles in the data collection were generated for systematic review. However, after considering the inclusion and exclusion criteria, only 14 were used for the systematic review activities to determine compost quality. The eight articles contain complete information on the parameters that can be used, including moisture content, pH, C-Organic, and N-total, as well as macro- and microcomponents consisting of phosphorus (P), potassium (K), calcium (Ca), sulfur (S), iron (Fe), magnesium (Mg), zinc (Zn), manganese (Mn), and copper (Cu), as presented in Table 5.

According to the articles reviewed, the composting period ranged from 30 to 150 days. However, using organic materials such as EFB mixed with the liquid waste of palm oil, containing local microorganisms, can help accelerate the decomposition of organic matter, thereby reducing the time. The period required to reach the maturity phase of the compost takes approximately 60 days, in line with the studies presented in Table 6.

A total of 14 parameters were analyzed, 6 of which had a significant result on pH, C-organic, C/N ratio, and elements such as P, K, Fe, Zn, Mn, and Cu. Moreover, some parameters in the heterogeneity test had a value >48%, except K, Ca, S, and Mg.

The bias in the basic parameters Ca, S, and Mg was caused by excessive intervention in small-sized studies with low methodological quality. The insignificant nature of these parameters does not mean that the results were not good. However, it showed that the expected decrease and increase in specific parameters could provide quality following the requirements and standards set.

Microorganisms need C-organic as a source of energy in the composting process. Hence, the longer the composting time, the lower the C-organic levels. It is because the presence of C carbon is required for microbes to breed [60]. C-organic content based on compost quality standard SNI 19-7030-2004 (Indonesia) ranges from 9.8% to 32%. In addition, microorganisms in the composting process also require a certain amount of N. The higher the total content, the faster the organic matter will decompose.

Phosphorus and potassium are the main nutritional elements/macronutrients in microbial compost. Based on the literature review, the phosphorus and potassium levels in the compost are 0.99% and 4.14%, respectively. Given that both are macrocomponents, the higher the percentage of P and K, the better the quality of the end product. Besides, nitrogen also influences phosphorus levels, indicating that the greater the N content, the higher the P due to the multiplication of remodeling microorganisms [85]. Microorganisms use potassium as a catalyst in the substrate material. The presence of bacteria and their activities will significantly cause an increase in the content [85]. Phosphorus plays a role in the forming of flowers and fruits. At the same time, potassium helps to develop and strengthen the tissue on the fruit stalk, thereby reducing the tendency to fall.

Calcium is another essential element for growth and makes plants less susceptible to disease and pests. However, pH strongly influences its availability, implying that soil with low pH tends to have poor phosphorus and calcium contents [67]. The calcium deficit is linked to a low soil pH, organic matter, and sandy soil texture. Moreover, applying palm oil liquid waste to land can increase the magnesium value [67]. Generally, roots absorb nutrients at a neutral pH quickly because they are readily soluble in water. In acid soils, toxic elements are found due to the increase in the solubility of microelements such as Fe, Mn, Zn, Cu, and Co in large quantities, which are toxic to plants. Soil pH also determines the development and population of soil microbes. Hence, bacteria and fungi beneficial to soil and plants will breed at pH > 5.5, but when the value is low, it leads to reduced growth and hampered development [67].

Mn, Zn, and Cu micronutrients are needed in the plant growth process at moderate amounts, but based on the review results, these microelements are still below the required limits, namely, 115.44 ppm, 443.18 ppm, and 52.50 ppm, respectively. Hence, they were classified as safe. Cu is an essential nutrient for plant growth needed in low concentrations because excessive amounts cause phytotoxicity. One of the organic materials that can reduce soil phytotoxicity is organic metals' complexity [86].

Due to its high affinity towards organic matter, Cu is not quickly mobilized in the composting process [26]. During the composting of solid-liquid waste, the formation of humic substances converts Cu from the humic substances to the organic fraction [87]. The cellular fraction decreases with increasing humic content and reducing pH from 7.5 to 6.7 in line with the affinity of Cu for organic matter [88]. The amount of adsorbed Cu decreased with an increase in the amount of C-organic dissolved in the substrate because the metal formed a stable complex that tended to remain in the substrate (Singh & Kalamdhad). Zn is also an element that is needed in small amounts, and its mobile fraction increases during the composting process due to the oxidation of organic matter and high oxidation-reduction potential. The formation of humate potentially converts Zn from the fraction of the humic substance to organic during the composting process [26].

Furthermore, Mn is a nutrient needed by plants in small amounts. The nutrient plays a critical role in synthesizing chlorophyll, a coenzyme, and acts as an activator of several respiratory enzymes in metabolic and photosynthetic reactions. A research study [26] reported that the availability of Mn decreased during the composting with animal manure.

Other metal elements such as Ni, Pb, Cd, and Cr in the compost are deficient and not expected to be present. The indicated heavy metal concentration was below the toxicity level, and Ni was not identified in the compost. After 40 days, Ni < 20 ppm, and when palm oil liquid waste was added, the concentration of heavy metals decreased in the final product [7]. The slight decrease might be related to the stabilization phase, where the moisture increased in the final stage.

### 3.6. Methods of Palm Oil Waste Application

Several methods are related to applying palm oil waste in the regulations and provisions set by the government in its utilization. The land application method tends to consider field-specific conditions consisting of eight main factors which are as follows: (i) the type and quantity of available waste, (ii) the shape of the ground surface, (iii) the soil type and groundwater depth, (iv) the area and distance from the WWTP (waste water treatment plant), (v) the distance between the area and the water source, (vi) the investment, operational, and maintenance costs, and (vii) the distance to residential areas. Meanwhile, the most common methods used in palm oil plantations are flat or long beds, furrows, sprinklers, and the tanker system. The differences between the methods are presented in Table 7.

Aside from developing composting technology, the following is an application review of composted EFB, POME, and other solid wastes as organic fertilizers for plant growth and productivity. According to several reviews, mulching is the most commonly used technique (Tables 8–10).

The solid waste application method generally uses a direct mulching system, either conventional or mechanical. Mulching is the practice of applying a layer of material to the soil surface to reduce temperature and increase pH and nutrients to improve plant growth and yield [95]. Using EFB mulching can effectively enhance soil aggregation and water retention. Furthermore, it facilitates weed control, prevents erosion, and helps maintain soil moisture, specifically for young palm oil [96]. Mulching can improve soil fertility because it contains the elements needed to improve the quality [53].

Liquid waste (POME) is generally applied directly as mulching in land application systems with various doses either through pond systems or biopori. This application can increase soil CEC up to 9.47 cmol·kg^−1^ [67], improving the chemical properties of ultisol soils [97]. The summary is presented in Table 9.

The compost from co-composting EFB and POME is generally used as organic compost with a mulching application method (Table 10). Several research results show that the mulching method still does not provide consistent results on plant productivity.

## 4. Conclusions

Optimal palm oil waste management is usually carried out using composting and considered as a sustainable technology because it is aimed at environmental conservation and providing economic value. The in-vessel method with CECC is the best system with the shortest period of 14 days. However, it requires extra management control and produces a final product with a high microorganism value compared to the outdoor and indoor windrow methods. Furthermore, the systematic review results on the compost quality of the 14 measured parameters, namely, humidity, pH, C-organic, N-total, and macro- and micronutrients such as P, K, S, Ca, Mg, Fe, Mn, Cu, Zn, and B which were still following the standards and requirements of compost quality in Indonesia. The palm oil waste application has advantages and disadvantages. The advantages include sorting from the top based on the most applied solid and liquid waste method, using a method with the furrow system, long bed, sprinkler, and tanker. The use of solid waste for direct fertilization (EFB) and compost through a manual or mechanical mulching system is placed on the soil surface between the palms. In another method, EFB is applied after composting through mulching to supply nutrients in stock nutrition for both soil and plants to improve the quality. However, a sustainable practice needs to be studied further for a better consistent result on the yield. Based on the systematic review, we found that there are still research biases and the need for finding new methods so that the quality of the compost produced from composting EFB and POME or other waste becomes organic fertilizer that can increase soil fertility, nutrient stock, and plant productivity sustainably [104].

## Figures and Tables

**Table 1:** Definitions of composting obtained from several references.

Compost definition	Reference
Composting is the controlled biological decomposition of organic waste into humus. It is also known as the bioconversion of organic waste substrates into stable end products (compost)	[21]
Composting is a solid phase of aerobic biodegradation through a natural heating process of organic matter under controlled conditions that distinguishes it from ordinary decay	[22]
Composting is a viable way to convert various organic wastes into products that can be safely used as biofertilizers and soil conditioners	[23]
Compost is the final product of decomposed organic plant and animal waste	[24]
Composting is the biological decomposition of organic matter under aerobic conditions	[25]
Composting is the biological decomposition and stabilization of organic substrates when a thermophilic process occurs due to a naturally generated heat to produce a stable final product that is free from pathogenic bacteria and can be applied to agricultural land	[26]
Composting is a viable way to convert various organic wastes into products that can be safely used as biofertilizers and soil conditioners	[27]
Composting is a biological decomposition and stabilization of organic waste that occurs naturally	[28]
Composting is the process of breaking down organic matter through the process of decomposition and digestion in an oxygen-rich (aerobic) environment	[29]

**Table 2:** Development of palm oil waste management.

Substrate (waste type)	Management (development technology)	Results	References
Liquid waste	Organic liquid fertilizer (fermentation using the EM4 activator)	Decreased COD from 2.362 mg/l to 1.580 mg/l and some of the organic matter was converted into new bacterial cells. The resulting product contains nutrients similar to organic fertilizers	[47]
EFB	Compost	The compost produced had the characteristics of pH 9, moisture content 57.24%, water holding capacity 76%, C/N ratio 12.5%, P 0.58%, and K 0.95% with a composting period of 40 days	[48]
EFB	Compost (mixture using palm oil mill effluent)	Compost nutrition is similar to other studies. In-vessel systems such as rotating drum composters are the systems with the fastest degradation of EFB -liquid waste of palm oil substrates	[49]
EFB	Compost (the addition of mud to the decanter and chicken manure)	Compost produced by standard nutrition	[50]
EFB	Compost (mixture of palm oil mill effluent and earthworm *Eisenia fetida*)	Good compost content on vermicompost is EFB with the addition of liquid waste of palm oil (50% EFB + 50% liquid waste of palm oil)	[51]
EFB	Compost (the addition of palm oil mill effluent anaerobic sludge)	Anaerobic liquid waste of palm oil. Anaerobic accelerates the composting period in 40 days with a C/N ratio of 12.4	[52]

Bunch fiber cell	(i) Nucleus plantation fertilizer (mulching)	100% EFB is used for land applications, and 100% fiber is used as fuel in the boiler. Refusing and reducing the system can save costs and increase company revenue	[53]
	(ii) Plasma plantation fertilizer (mulching)		
	(iii) Partnership plantation fertilizer (mulching)		
	(iv) Community plantation fertilizer (mulching)		
	(v) Boiler energy		
	(vi) Boiler surplus		
	(vii) Dryer fuel		

Liquid waste	Land application	Applying the thermophilic system to the anaerobic reactor unit might increase the decomposition of bacteria, thereby making the processing more efficient	[54]
Liquid waste	Biogas production (pilot-scale closed pond system with semi and continuous feeding)	The liquid waste of palm oil processing was stable at an organic loading rate of 0.9–3.11 kg/m^3^/day with a COD reduction percentage of more than 90% and a maximum biogas production of 2.59 m^3^/day	[55]
Liquid waste	Reduce odor (flotation system)	Flotation of palm oil's liquid waste effectively reduces fat/oil content, COD, MLSS, and MLVSS. The longer the period of residence of the liquid in the flotation, the higher the decrease	[39]

**Table 3:** Comparison between windrow-based and CECC environment [62].

Parameter	Outdoor system	Indoor system	CECC
Compost location	Open air >10 Ha	Indoor shelter >4 Ha	Controlled closed environment
Weather protection	Compost cover and no rainwater puddle control	Shelter/roof	In controlled room
Operational complexity; compost period	Supervision manually; 60–90 days	Manual supervision; 45–60 days	Computer controlled; 14 days
Moisture addition	Not controlled (weather)	Not completely controlled	Fully controlled
Temperature control	No	No	Fully controlled
Leachate control	Minimum	Fully	Controlled and recyclable
Quality control and traceability	No	Part	Fully
Production of bio-organic fertilizer	Noncompliance and low-quality compost only	Partial compliance, inconsistent quality, and colony form unit	Full compliance and high colony form unit calculation

**Table 4:** Comparison of review data of compost standards based on the Indonesian standards (SNI 19-7030-2004) and Minister of Agriculture (Regulation No. 261/KPTS/SR.310/M/4/2019).

Parameter	SNI 19-7030-2004	Permentan no 261/KPTS/SR.310/M/4//2019	Review results
C-organic (%)	9.80–32	min 15	—
C/N ratio	10–20	≤25	23.42
pH	6.80–7.49	4–9	7.41
Temperature (°C)	Groundwater temperature	—	Variation (in standard)
Water content (%)	Max. 50	8–20	—
Macroelements:			—
N-total (%)	Min. 0.4	Total = Min. 2	—
Phosphorus (%)	Min. 0.10		0.99%
Potassium (%)	Min. 0.20		4.14%
Heavy metal (ppm):			
As	—	Max. 10	—
Hg	Max. 0.8	Max. 1	—
Pb	Max. 150	Max. 50	—
Cd	Max. 3	Max. 2	—
Cr	Max. 210	Max. 180	—
Co	Max. 34	—	—
Cu	Max. 100	—	52.50 ppm
Ni	Max. 62	Max. 50	—
Se	Max. 2	—	—
Microelements:			
Ca	Max. 25.50%	—	1.29%
Mg	Max. 0.60%	—	0.7%
Fe total	Max. 2.00%	Max. 15000 (ppm)	0.78%
Fe available		Max. 500 (ppm)	
Mn	Max. 0.10%	—	115.44 ppm
Zn	Max. 500 (ppm)	Max. 5000 (ppm)	442.18 ppm
Al	Max. 2.20%	—	—

**Table 5:** The analysis results on physicochemical parameters and macro- and micronutrient content of palm oil waste compost.

Parameter	*N*	*k*	SMD	*p*	*I* ^2^(*p*)	Egger′s (*p*)
Physicochemical						
Moisture	75	8	−0.946 [−1.440; −0.452]	<0.001	93.40% (<0.001)	0.039
pH	95	8	0.968 [0,520; 1.415]	<0.001	48.54% (0.015)	0.001
C-organic	135	8	0.926 [0.572; 1.280]	<0.001	92.90% (0.003)	0.001
N-total	135	8	2.19 [1.762; 2.616]	<0.001	93.35% (<0.001)	<0.001
C/N ratio	120	8	0.407 [0.029; 0.785]	0.035	82.28% (<0.001)	0.001
Macro- and microcomponents						
P	125	8	0.698 [0.321; 1.076]	<0.001	60.51% (0.118)	<0.001
K	125	8	0.847 [0.464; 1.230]	<0.001	35.41% (0.242)	<0.001
Ca	70	8	−57.6 [−67.191; −48.072]	<0.001	0% (0.557)	ns
Mg	95	8	−0.212 [−0.641; 0.217]	ns	34.46% (0.267)	ns
S	18	8	−0.33 [−1.34; 0.68]	ns	10.18% (0.355)	0.155
Fe	24	8	0.99 [−1.32; 3.30]	ns	83.66% (0.033)	0.005
Zn	18	8	5.64 [2.36; 8.91]	<0,001	52.94% (0.047)	0.013
Mn	18	8	1.33 [−2.20; 4.85]	ns	88.75% (0.007)	0.008
Cu	24	8	3.38 [0.64; 6.12]	0.016	76.45% (0.003)	<0.001

Description: *N* = number of samples used in the study; *k* = number of studies used in the analysis; SMD = standardized mean difference is a summary of each study with the effect size; *p* = *p* value; *I*^2^ = inconsistency (heterogeneity test); Egger's = publication bias test; ns = not significant.

**Table 6:** Physicochemical data of compost from several studies.

Ref	Material	Time (Day)	Temperature (°C)	Moisture (%)	pH	C/N	P	K	S	Ca	Mg	Fe	Mn	Cu	Zn	B
[68]	EFB	60	27–35	47–57	5.2	68.39	*n*	*n*	*n*	*n*	*n*	*n*	*n*	*n*	*n*	*n*
	EFB and EM4	60	30–44	45–65	5.9	29.80	*n*	*n*	*n*	*n*	*n*	*n*	*n*	*n*	*n*	*n*
	EFB and local decomposer vegetable waste	60	30–54	50–65	6.9	21.18	*n*	*n*	*n*	*n*	*n*	*n*	*n*	*n*	*n*	*n*
	EFB and local decomposer	60	30–52	54–65	6.2	33.37	*n*	*n*	*n*	*n*	*n*	*n*	*n*	*n*	*n*	*n*
	Banana hump EFB and palm oil liquid waste	60	30–54	50–65	6.7	24.10	*n*	*n*	*n*	*n*	*n*	*n*	*n*	*n*	*n*	*n*

[11]	EFB and chicken manure	45	*n*	*n*	*n*	28.03	*n*	*n*	*n*	*n*	*n*	*n*	*n*	*n*	*n*	*n*
	EFB, chicken manure, and inoculum 1	45	*n*	*n*	*n*	19.63	*n*	*n*	*n*	*n*	*n*	*n*	*n*	*n*	*n*	*n*
	EFB, chicken manure, and inoculum 2	45	*n*	*n*	*n*	17.27	*n*	*n*	*n*	*n*	*n*	*n*	*n*	*n*	*n*	*n*
	EFB, chicken manure, and inoculum 3	45	*n*	*n*	*n*	16.68	*n*	*n*	*n*	*n*	*n*	*n*	*n*	*n*	*n*	*n*

[69]	EFB and palm oil liquid waste	30	30–40	70–75	8.5	17.95	*n*	*n*	*n*	*n*	*n*	*n*	*n*	*n*	*n*	*n*
		30	30–40	70–75	8.5	14.13	*n*	*n*	*n*	*n*	*n*	*n*	*n*	*n*	*n*	*n*
		30	30–40	70–75	8.5	28.41	*n*	*n*	*n*	*n*	*n*	*n*	*n*	*n*	*n*	*n*

[70]	EFB	60	*n*	*n*	*n*	17.92	0.5	6.1	*n*	*n*	*n*	*n*	*n*	*n*	*n*	*n*
	EFB and *M. bracteata*	60	*n*	*n*	*n*	14.39	0.6	4.2	*n*	*n*	*n*	*n*	*n*	*n*	*n*	*n*
	EFB and *M. bracteata*	60	*n*	*n*	*n*	12.30	0.6	4.9	*n*	*n*	*n*	*n*	*n*	*n*	*n*	*n*
	EFB and *M. bracteata*	60	*n*	*n*	*n*	14.43	0.6	6.6	*n*	*n*	*n*	*n*	*n*	*n*	*n*	*n*

[71]	EFB and palm oil liquid waste	52	29.73	72.03	6.71	29.45	*n*	3.8	*n*	*n*	*n*	*n*	*n*	*n*	*n*	*n*
	EFB, palm oil liquid waste, fish pellets, bone pellets, ash, and sawdust	52	28.30	59.32	6.32	16.01	1.2	12	*n*	*n*	*n*	*n*	*n*	*n*	*n*	*n*
	EFB, palm oil liquid waste, fish pellets, bone pellets, ash, and sawdust	52	28.10	72.44	6.41	15.15	1.3	12	*n*	*n*	*n*	*n*	*n*	*n*	*n*	*n*
	EFB, palm oil liquid waste, fish pellets, bone pellets, ash, and sawdust	52	28.13	64.71	6.17	22.33	2.7	12	*n*	*n*	*n*	*n*	*n*	*n*	*n*	*n*

[9]	EFB, palm oil liquid waste, and perlakuan 1	60	55	67	9.08	*n*	0.2	6.0	*n*	*n*	0.4	*n*	*n*	*n*	*n*	*n*
	EFB, palm oil liquid waste, and perlakuan 2	60	55	67	8.75	*n*	0.3	3.7	*n*	*n*	0.4	*n*	*n*	*n*	*n*	*n*
	EFB, palm oil liquid waste, and perlakuan 3	60	55	70	8.50	*n*	0.3	3.8	*n*	*n*	0.5	*n*	*n*	*n*	*n*	*n*

[72]	EFB	64	26–52	*n*	8.12	63.32	0.2	8.2	*n*	8	2.5	0.9	*n*	0.03	*n*	0.3
[73]	EFB and palm oil liquid waste	14	27–30	*n*	8.2	25	*n*	*n*	*n*	*n*	*n*	*n*	*n*	*n*	*n*	*n*
		14	27–30	*n*	8.0	30	*n*	*n*	*n*	*n*	*n*	*n*	*n*	*n*	*n*	*n*
		14	27–30	*n*	8.1	35	*n*	*n*	*n*	*n*	*n*	*n*	*n*	*n*	*n*	*n*
		14	27–30	*n*	8.0	40	*n*	*n*	*n*	*n*	*n*	*n*	*n*	*n*	*n*	*n*
		14	27–30	*n*	8.0	45	*n*	*n*	*n*	*n*	*n*	*n*	*n*	*n*	*n*	*n*
		14	27–30	*n*	8.2	50	*n*	*n*	*n*	*n*	*n*	*n*	*n*	*n*	*n*	*n*
		14	27–30	*n*	8.2	55	*n*	*n*	*n*	*n*	*n*	*n*	*n*	*n*	*n*	*n*

[74]	EFB and *Streptomyces* sp.	60	*n*	*n*	*n*	20.4	0.7	1.8	*n*	*n*	*n*	*n*	*n*	*n*	*n*	*n*
	EFB and *Bacillus* sp.	60	*n*	*n*	*n*	18.2	0.6	1.8	*n*	*n*	*n*	*n*	*n*	*n*	*n*	*n*
	EFB and *Phanerochaete chrysosporium*	60	*n*	*n*	*n*	17.5	0.8	1.9	*n*	*n*	*n*	*n*	*n*	*n*	*n*	*n*
	EFB, *Streptomyces* sp., and *Bacillus* sp.	60	*n*	*n*	*n*	16.4	0.8	1.9	*n*	*n*	*n*	*n*	*n*	*n*	*n*	*n*
	EFB, *Streptomyces* sp., *Bacillus* sp., and *Phanerochaete chrysosporium*	60	*n*	*n*	*n*	16.1	0.9	2	*n*	*n*	*n*	*n*	*n*	*n*	*n*	*n*
	EFB and palm oil liquid waste	60	*n*	*n*	*n*	21.3	0.7	2	*n*	*n*	*n*	*n*	*n*	*n*	*n*	*n*

[75]	Fresh palm oil, chicken manure, and rice washing water	21	56	40–60	*n*	15.79–21.34	*n*	*n*	*n*	*n*	*n*	*n*	*n*	*n*	*n*	*n*
[76]	EFB, leaf, and chicken manure	80	49–57	40–65	7.15–7.38	17.6–26.1	*n*	*n*	*n*	*n*	*n*	*n*	*n*	*n*	*n*	*n*
[77]	Fresh palm oil	60	56	50–70	8.2	18	0.1	0.9	0.4	0.6	0.2	0.2	72	24	38	9
[78]	Palm oil and biogas waste	60		38.35	6.92	8.17	*n*	*n*	*n*	*n*	*n*	*n*	*n*	*n*	*n*	*n*
	Palm oil, biogas waste, and EFB	60	44.66	47.35	7.82	15.77	*n*	*n*	*n*	*n*	*n*	*n*	*n*	*n*	*n*	*n*
	Palm oil biogas waste and decanter cake	60	*n*	50.70	7.75	7.57	*n*	*n*	*n*	*n*	*n*	*n*	*n*	*n*	*n*	*n*
	Palm oil biogas waste, EFB, and decanter cake	60	49	43.77	7.79	13.47	0.9	*n*	*n*	*n*	*n*	*n*	*n*	*n*	*n*	*n*

[7]	EFB and palm oil liquid waste	40	67	55–65	8.1	12.4	1.4	2.8	0.2	1.0	0.9	1.0	151	74	157	11
[79]	EFB and palm oil liquid waste	70	70	*n*	7.7	15	4.5	80	*n*	10	13	*n*	*n*	38	154	*n*
[34]	EFB, palm oil liquid waste, and decanter cake	51	79	50–60	8.527	18.6	1.2	2.9	*n*	1.2	0.8	*n*	*n*	*n*	*n*	*n*
	EFB and palm oil liquid waste	51		50–60	8.627	28.02	1	2.5	*n*	0.9	2.5	*n*	*n*	*n*	*n*	*n*

[80]	EFB and palm oil liquid waste	60	35.3	60	7.8	12.8	*n*	*n*	*n*	*n*	*n*	*n*	*n*	*n*	*n*	*n*
[81]	EFB and palm oil liquid waste	60	35	61	8.1	12.7	1.3	2.8	1.1	0.7	0.9	1.2	245	68	87	*n*
[82]	EFB and sewage sludge (4:1)	84	28–41.5	*n*	6.9	22.16	0.5	2.5	*n*	0.4	0.3	0.5	99	67	723	*n*
	EFB and sewage sludge (3:1)			*n*	6.7	21.83	0.6	4	*n*	0.4	0.4	0.7	108	68	881	*n*
	Frond and sewage sludge (4:1)			*n*	6	24.6	0.8	2	*n*	0.5	0.2	0.5	85	52	495	*n*
	Frond and sewage sludge (3:1)			*n*	5.8	29.67	1	2.2	*n*	0.5	0.2	0.5	98	53	675	*n*
	Trunk and sewage sludge (4:1)			*n*	6.2	18.98	0.6	1.4	*n*	0.7	0.2	0.6	93	69	671	*n*
	Trunk and sewage sludge (3:1)			*n*	6.1	19	0.9	1.7	*n*	0.6	0.3	0.7	88	79	829	*n*

[83]	Palm oil liquid waste and sawdust	300	40	*n*	5.7	19	*n*	*n*	*n*	*n*	*n*	*n*	*n*	*n*	*n*	*n*
[84]	EFB and palm oil liquid waste	98	70–75	*n*	7.5	15	3.1	55		15	9.6	*n*	*n*	38	154	0.1

Description: n = none (not available): P = phosphorus, K = potassium, S = sulfur, Ca = calcium, Mg = magnesium, and Fe = iron are represented by the unit % (percentage); Mn = manganese, Cu = copper, Zn = zinc, and B = boron are represented by the units ppm or mg/kg.

**Table 7:** Comparison of palm oil effluent methods [89].

Application method	Strengths	Weaknesses
Flatbed or long bed system	(i) Higher capacity compared to the furrow system	(i) Investment and operational costs are more expensive than the furrow system
	(ii) Suitable use in sloping/relatively flat hilly areas	(ii) Can damage palm oil trees if overfeeding is done
	(iii) Equitable distribution of waste and good soil element storage	(iii) Complicates the work of harvesters compared to furrows
	(iv) Less soil erosion effect	(iv) More pungent smell
	(v) Rarely clogged	
	(vi) Low investment cost compared to sprinklers	

Furrow system	(i) At the same volume, less area is required	(i) Affected by soil erosion
	(ii) Cheaper investment costs compared to sprinklers and long beds	(ii) Easy to clog and overflow
	(iii) The potential for damaging palm oil trees is more diminutive than long beds	(iii) Need control during effluent irrigation
	(iv) Suitable use in areas with clay or clay soil types	(iv) Operating costs are higher than long beds
	(v) Easier to maintain	
	(vi) Do not complicate harvesters	
	(vii) Increase in yield/ha/year is almost the same as sprinklers and long beds	
	(viii) Relatively less smell than a long bed	

Sprinkler system	(i) Sufficiently high waste delivery capacity	(i) High investment and operational costs
	(ii) Can distribute liquid waste over a large area	(ii) Need more maintenance
	(iii) Not affected by the contours of the land	(iii) Pipes can be clogged and broken
	(iv) Not affected by soil erosion	(iv) The sprinkler can get stuck due to blockage

Tanker system	(i) Cheaper investment and operational costs than other systems	(i) Potential for pollution outside the area is relatively high
	(ii) Can apply liquid waste in areas far from WWTP	(ii) Lower effluent application capacity
		(iii) Influenced by the condition of road and bridge infrastructure in the application area
		(iv) Influenced by the season
		(v) Affected by the condition of the tractor/tanker
		(vi) Application of liquid waste at any time can be stopped entirely
		(vii) Easy to clog and overflow
		(vii) Need control during waste watering

**Table 8:** Review of the development of the EFB compost application.

Methods	Dosage	Results	References
Mulching with planting beds with the size of 2.6 m × 0.8 m with a distance of 0.5 m (planting holes 0.6 × 0.6 m)	20–60 ton/ha	The application of 60 tons of EFB compost was the highest yield in young mature compared to other treatments in the postmining land. The application of EFB compost also reduced the increase in metals, specifically Pb	[90]

Mulching	1.5–2 kg/plot	The application of several doses of EFB compost showed significant differences in all parameters: plant height, number of leaves, leaf area, root volume, and fresh weight of crop consumption. The dosage of 1.5 kg plot^−1^ to 2 kg plot^−1^ showed the best growth and production in mustard plants	[91]

Mulching (soil mixing of growing media)	1:2 (compost: growing media)	The addition of leaves on chili and corn plants with EFB compost was faster than urea and ordinary soil, while the weight of plants with compost was greater than urea and soil	[92]

Mulching in inceptisol soil pH 5.2	3–12 ton/ha	The administration of several doses of EFB compost showed different effects on the parameters of plant height, plant fresh weight, and fresh weight of plants fit for consumption. The application of EFB compost at a dose of 9 tons/ha is the best dose for producing pakcoy plants	[93]

Mulching (soil mixing growing media)	37.5 g–112. 5 g per polybag with the size of 35 cm × 40 cm	The combination of EFB compost significantly affected seedling height, number of leaves, root volume, root crown ratio, and dry weight of 4 months old palm oil seedlings. The best treatment was shown by the combination of 112.5 g per polybag EFB compost with 18 and 27 g per polybag dolomite on inceptisol soil	[94]

**Table 9:** Review of the development of POME applications.

Methods	Dosage	Results	References
Randomly sampling on peat soil (soil analysis)	@700 g mixed with the liquid waste of palm oil	There was a change in composition for the pH value and mineral content of the peat soil after the application of POME with a pH value of 6.20, C-organic 2.38%, N-total 0.28%, phosphorus 63.34 ppm, Calcium 2.40 cmol/kg, magnesium 1.37 cmol kg^−1^, potassium 1.06 cmol kg^−1^, and CEC 9.47 cmol kg^−1^.	[67]

Biopore (palm oil mill effluent from an aerobic pool)	5; 7.5; and 10 liters of liquid waste of palm oil are provided in 1, 2, 3, and 4 biopore/plant holes, respectively	The application of POME dose of 7.5 liters in four biopores/plant holes increased the number of midribs and leaves, leaf width and length compared to the control and the highest form of other treatments.	[98]

Mulching with the size of 1.2 × 1.4 m with plant spacing 60,70 cm.	100, 200, and 300 ml per plan Mixed crops with palm ash	The interaction of POME and ash significantly affected plant height and weight of fruit/plant with the best dose of giving POME 300 ml with a fruit weight of 670 g per plant.	[99]

Mulching plot size 4 × 4 m with a spacing of 20 × 40 cm)	25000, 50000, 75000, 100000, and 150000 L/ha	Applying 150000 L ha^−1^ POME can replace dolomite lime, manure, urea fertilizer, and KCl in improving the chemical properties of ultisol soil for soybean production.	[97]

**Table 10:** Review of the development of soil-mixed waste applications.

Methods	Dosage	Results	References
Mulching (three same-age plant blocks)	POME and EFB as organic fertilizers	The POME application produced an FFB that was not significantly different from the EFB application although the production of the EFB block was lower than the POME block. The average FFB production in both blocks was already above the potential for moderate-class land production. The POME application resulted in higher plant height, female flowers, and sex ratio than the EFB application	[100]

Mulching (three blocks with a total of 60 samples)	POME and EFB as organic fertilizers	Applying organic fertilizer had the same effect on palm oil plants' production and agronomic character. Palm oil production on land where EFB and POME were applied was still below the production potential according to the intermediate class of land production	[101]

Mulching (three blocks with a total of 60 samples)	POME and EFB as organic fertilizers	Each year's growth and productivity of palm oil plantations had not yet reached the production potential in the moderate-class of land suitability. POME and EFB applied the same effect on all agronomic characteristics of palm oil plant growth	[102]

Mulching	Composition of planting media (sand soil, clay soil, and a mixture of both) with the addition of four doses of dry mud (% volume) with 10%, 20%, 30%, and 40% seeds	There was no excellent combination between the dosage of POME dry mud and soil type. Applying dry mud POME concentration of 10% was sufficient to produce good growth of palm oil seedlings. The four doses gave the same effect as NPK fertilizer and urea at a dose of 0.4 g on palm oil growth	[103]

## Data Availability

The experimental data used to support the findings of this study are available from the corresponding author upon request. The data are also available at https://fairsharing.org/users/6709.
